# SARS-CoV-2 Reinfection among Healthcare Workers in Mexico: Case Report and Literature Review

**DOI:** 10.3390/medicina57050442

**Published:** 2021-05-03

**Authors:** Brenda Garduño-Orbe, Juan Manuel Sánchez-Rebolledo, Mustafá Cortés-Rafael, Yuliana García-Jiménez, Marcelina Perez-Ortiz, Indira Rocío Mendiola-Pastrana, Eduardo López-Ortiz, Geovani López-Ortiz

**Affiliations:** 1 Unidad de Medicina Familiar Número 26, Instituto Mexicano del Seguro Social, 39700 Acapulco, Guerrero, Mexico; brendaorbe@gmail.com (B.G.-O.); sanchezrebolledoaca@yahoo.com (J.M.S.-R.); yulianagaji1981@gmail.com (Y.G.-J.); mercipam_11@hotmail.com (M.P.-O.); 2 Hospital General Regional No. 1 Vicente Guerrero, Instituto Mexicano del Seguro Social, 39610 Acapulco, Guerrero, Mexico; mustafacortes@gmail.com; 3 Subdivisión de Medicina Familiar, Facultad de Medicina, Universidad Nacional Autónoma de México, 04510 Ciudad de México, Mexico; dramendiolapastrana@gmail.com (I.R.M.-P.); eduardolptz@gmail.com (E.L.-O.)

**Keywords:** COVID-19, reinfection, SARS-CoV-2, healthcare workers, clinical reinfection

## Abstract

Since the onset of the COVID-19 pandemic, there have been multiple questions regarding reinfections associated with SARS-CoV-2. Healthcare workers on duty, due to overexposure in environments where there are more cases of COVID-19, are more prone to become infected by this virus. Here, we report 4 cases that meet the definition of clinical reinfection by SARS-CoV-2, as well as a literature review on this subject; all occurred in healthcare workers in Acapulco Guerrero, Mexico who provide their services in a hospital that cares for patients with COVID-19. The time between the manifestation of the first and second infection for each case was 134, 129, 107 and 82 days, all patients presented symptomatology in both events. The time between remission of the first infection and onset of second infection was 108, 109, 78 and 67 days for each case, while the time to confirmation by reverse transcription polymerase chain reaction (RT-PCR) between infections was 134, 124, 106 and 77 days. In two of the four cases the reinfection resulted in a more severe case, while in the remaining two cases the manifestation of symptoms and complications was similar to that presented in the first infection. Given this scenario, greater care is needed in the management of the pandemic caused by SARS-CoV-2 to protect healthcare workers and the general public from risks and complications caused by a possible reinfection by SARS-CoV-2.

## 1. Introduction

The first reports of infected persons by SARS-CoV-2 in Mexico occurred in the last week of February 2020; on 18 March 2020, it was confirmed the first death in the country due to complications associated with COVID-19; since the beginning of the pandemic in Mexico, there have been reports of high fatality rates compared to other parts of the world [1]. As of 23 March 2021, according to official reports, there have been a total of 2,397,991 accumulated cases and 199,048 deaths; since May 2020, the country has registered hundreds of deaths per day due to this disease; its peak occurred on 11 February 2021 with a weekly average of 1428 deaths [2,3]. 

Health professionals, due to overexposure to environments with a high level of contagion, represent one of the highest risk groups of becoming infected by SARS-CoV-2 [4,5]. 

Thousands of front-line physicians and healthcare workers have died due to complications caused by COVID-19, many of the deaths have occurred in low- and middle-income countries [5]. 

In Mexico, since the beginning of the pandemic, the number of infected healthcare workers amounts to about 200,000, it has also been reported that it is the country with the highest number of deaths among healthcare workers (>3000), where about 50% corresponded to physicians and almost 20% to nurses [6,7]. 

Because COVID-19 is a relatively new disease, several aspects of its progression and long-term health effects are unknown, one of the aspects that have become more relevant as time goes by is the impact that reinfections have on health [8].

To date, the number of reinfections is marginal in relation to the number of cases of COVID-19; to systematize different reinfection scenarios, operational algorithms have been established to define such cases [9,10]. In this article, it is reported the existence of 4 cases of clinical reinfection by SARS-CoV-2 in healthcare workers in Mexico.

## 2. Methods

Case report study in 4 healthcare workers, meeting the definition of clinical reinfection with SARS-CoV-2, serving in a hospital caring for patients with COVID-19 in Acapulco Guerrero, Mexico. The study lasted from April to October 2020. Written informed consent was obtained from the patients for publication of this case. 

To detect the presence of SARS-CoV-2, SAR-CoV-2 RT-qPCR assays were performed with the SuperScript III Platinum One-Step qRT-PCR kit (ThermoFisher Scientific, Carlsbad, CA, USA), according to the manufacturer’s specifications. 

Available scientific information on SARS-CoV-2 reinfection was identified by a systematic PubMed search carried out in January 2021, using the terms COVID-19, SARS-CoV-2, and reinfection, which yielded 132 articles, of which 17 were included because of their informative content related to the topic in the title or abstract.

## 3. Case Reports

Case 1. 40-year-old female, nurse, with systemic arterial hypertension of 3 years of diagnosis, smoking of 11 years of evolution, at a rate of 3 to 4 cigarettes per day. On 26 April 2020, she started with fever of 38.7 °C, dry cough, and scarce nasal drainage, 3 days later she presented medium effort dyspnea, myalgia, arthralgia, increased cough and headache. On 29 April 2020, reverse transcription polymerase chain reaction (RT-PCR) was performed for SARS-CoV-2 in nasopharyngeal exudate, which reported positive. The patient developed exacerbation of symptoms and decreased oxygen saturation up to 84%, 7 days after the onset of the symptoms, accompanied by maculopapular rash on the upper and lower limbs, chest, face and neck, anosmia and dysgeusia. Twenty days after the beginning of symptoms, a chest X-ray was performed showing an increase in bronchial tram track, faint left basal opacity, without evidence of pneumonic data. The patient evolved to improvement and was reported asymptomatic 23 days after the onset of symptoms, so she was discharged and went back to work. On 7 September 2020, she presented a sudden general condition attack, sneezing, runny nose, myalgias, arthralgias, followed by fever, dry cough, headache, and dyspnea on medium exertion. On 10 September, symptoms increased in intensity, with a documented saturation of 90%, so RT-PCR for SARS-CoV-2 of nasopharyngeal exudate was performed again, and it was reported positive on September 11. On 18 September, a chest computerized tomography (CT) scan with lung window was performed, which reported increased diffuse opacity and central air cystic cavities, data of central-lobular emphysema of the right lung, with no data of alveolar involvement or tumors. On 26 September, she presented only sporadic cough and dyspnea on maximum effort, assessed by pulmonology with a diagnosis of post-infectious bullae. On 10 October 2020, she was discharged.

Case 2. 49-year-old female, nurse, with a 15-year history of hypothyroidism, no history of chronic degenerative pathologies, smoking denied. On 10 May 2020, she started with nasal congestion, myalgia, arthralgia, chills, headache, dry cough and dysgeusia. On 17 May, RT-PCR for SARS-CoV-2 of nasopharyngeal exudate was performed and reported positive on 19 May. Anosmia, diffuse fine maculopapular exanthema, and insomnia were added. She evolved favorably and was discharged on 30 May and returned to work. On 16 September 2020, she presented sudden onset of symptoms with headache, dry cough, odynophagia, myalgias, arthralgias, then dyspnea on medium efforts and conjunctivitis. On 18 September, RT-PCR for SARS-CoV-2 of nasopharyngeal exudate was performed again, which reported positive. A chest X-ray was reported with focal interstitial pattern predominantly right base and bilateral faint focal opacity, without evidence of pleural effusions. Treatment was given, she did not require hospitalization and she was discharged, finding the patient asymptomatic, on 21 October 2020.

Case 3. 53-year-old male, occupation: pharmacy assistant. With a history of pulmonary tuberculosis in 1988, for which he received complete treatment and was cured, with no history of chronic degenerative diseases, smoking denied. On 22 May 2020 he presented fever and dyspnea of medium efforts. On 25 May 2020, RT-PCR was performed for SARS-CoV-2 from nasopharyngeal exudate, which reported positive. On 1 June 2020, a chest X-ray was performed, and a ground-glass image, predominantly on the right basal, was reported, with bilateral faint focal opacity. Treatment was given, with adequate evolution. On 20 June 2020 he was discharged asymptomatic and without complications, so he returned to work. On 6 September 2020 he presented sudden onset of symptoms characterized by fever, chills, anosmia, dysgeusia, dry cough, rhinorrhea and general malaise. On 8 September 2020 RT-PCR for SARS-CoV-2 of nasopharyngeal exudate was performed again and was reported positive. Treatment was given again. He evolved with chest pain, pulse oximetry 97%, the chest X-ray reported faint focal opacity predominantly basal right, increased bronchial tract, with no data of pneumonia, on 22 September 2020. The presence of tuberculosis and other bacterial infections was ruled out by laboratory studies. He evolved to improvement with the established treatment. On 15 October 2020 he was discharged due to clinical criteria, and return to work.

**Figure 1:**
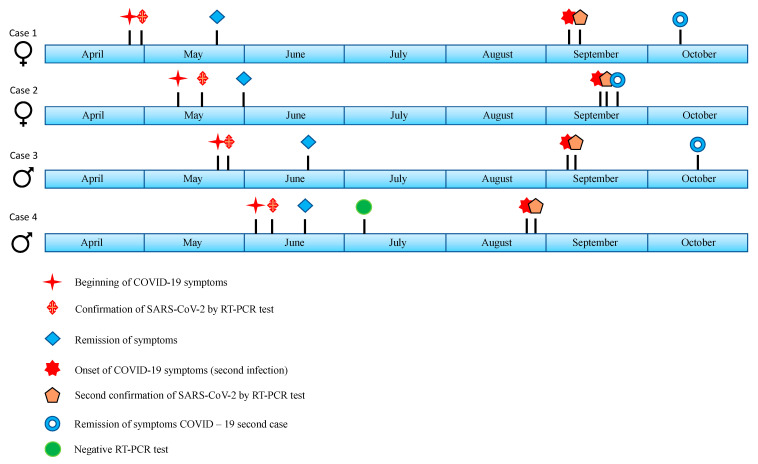
Timeline showing the most important clinical reinfection events in healthcare workers.

Case 4. 52-year-old male, occupation: internist. No pathological history, smoking denied. On 03 June 2020 he presented a sudden onset of illness with odynophagia and dry cough. On 11 June, a RT-PCR for SARS-CoV-2, from nasopharyngeal exudate, was performed and was reported as positive. Medical treatment was given, he did not require hospitalization, with no decrease in oxygen saturation. He was discharged on 18 June, and returned to work. On 10 July 2020, RT-PCR for SARS-CoV-2 was performed again, which was reported negative, so he was discharged and returned to work. On 24 August 2020, he suddenly presented myalgias, arthralgias, general condition attack, dry cough, dyspnea, odynophagia, and dyspnea of moderate efforts. Oxygen saturation of 81% was documented, requiring hospitalization. RT-PCR for SARS-CoV-2 of nasopharyngeal exudate was performed again on 27 August 2020, and was reported positive. He evolved with a decrease in pulse oximetry to 77%, despite supplemental oxygen intake, so he was admitted to intensive care, advanced airway management was started for 11 days. On 31 August, a simple chest CT scan was performed, which reported distribution of bilateral multilobar ground glass opacities, with areas tending to consolidate in the posterior segments of upper and lower lobes, interlobular septal thickening associated with ground glass mainly in the upper lobes of the right lung and atelectasis in the lower lobes, lingula and middle lobe. The patient evolved to improvement and was discharged on 17 November 2020. (Table 1, Figure 1).

## 4. Discussion

Since the beginning of the pandemic, there have been reports on the possibility of reactivation or reinfection by SARS-CoV-2, some of which reported, for both scenarios, an approximate time of no more than 15 days from the first medical discharge to the second confirmation or symptomatology [11,12,13]. Likewise, cases have been reported of persons who continue to be positive for SARS-CoV-2 up to 36 days after complete resolution of the disease [14]. In this context, it has been pointed out that extended periods of positive RT-PCR tests in patients could be associated with prolonged viral shedding. Other studies have reported an average time of 34.5 days between the first and second RT-PCR confirmation in possibly reinfected health professionals [15].

The presence of repeated infectious conditions caused by microorganisms considered monophasic and capable of generating immunity, or at least transitory, has led researchers to consider terms different from reinfection. In the specific case of COVID-19 infection and disease, the possibility of reactivation of a latent infection or relapse have been considered; however, there is no evidence that the infection remains latent in the organism. What remains, is the presence of non-replicative viral traces up to a maximum of 6 weeks after the onset of symptoms [12,16]. Conversely, the term recrudescence, which refers to the persistence of the infectious disease due to treatment failure [17], is not a term considered in the study and follow-up of COVID-19, however, not having a specific antiviral treatment could lead to consider this situation. Another important aspect in the evolution of the infectious conditions is given by the inflammatory rebound, which can be triggered by an inappropriate immune response, conditioning the recurrence of the symptoms [18]. Specifically, in the present study, recrudescence and inflammatory rebound are not considered due limited probability of occurrence, since the patients remained symptom-free for more than 60 days.

The emergence of new SARS-CoV-2 variants is inevitable due to biological processes such as selection pressures [19], a concern that arises due to the impact on the course of the COVID-19 pandemic [20]. In this context, reinfections could primarily be associated with strains different from those of a first infection; among the observations that support this hypothesis are: (1) genomic evidence: in the best characterized cases of reinfections, the two episodes have been caused by strains with different phylogenetic origins [21,22,23,24].; (2) deficient immune responses to new variants: it has been pointed out that new lineages of SARS-CoV-2 could evade immune responses acquired in past infections or reduce the capacity for neutralization by polyclonal antibodies [25,26], and (3) infectious behavior of other coronaviruses: the susceptibility to reinfection by new coronavirus strains (HCoV species), is much higher (9:1) than that of becoming infected by the same species [27]. Due to the limitations of viral sequencing in Mexico, it cannot be established that the reinfections reported in this study were associated with new variants; however, since the beginning of the pandemic, the country has been characterized by the introduction of SARS-CoV-2 variants [28]. Additionally, the lack of proper restrictions for travelers from other countries could have influenced the introduction and spread of new variants that could potentially cause the reinfection events.

While cases of reinfection by SARS-CoV-2 are still uncommon events, in comparison with the number of infected persons, more evidence has accumulated supporting this trend. It has been pointed out that reinfection caused by respiratory viruses, among the 4 human coronaviruses, may be due to weak or incomplete initial immune responses and cause a new infectious [10,29], these reinfection processes even with the presence of antibodies to pre-existing coronavirus may become common. On the other hand, the reactivation of infections, as well as the relapse or latency in the case of coronavirus is a subject of controversy and to date it has not been endorsed [9]. 

Reinfection by endemic human coronaviruses (no SARS-CoV-2) has been detected in an average of 6 months after the first infection, with a minimum of 50 days. Most of the times, these episodes occur with less intensity and lower viral titers, however, about 11% of the cases have shown to present a greater viral dissemination, compared to the previous infection. The second infection can then be of the same or greater intensity and it is probable that this second infection occurs by a new species of coronavirus [27,30,31]. Regarding SARS-CoV-2 infection, it has been shown that most people develop antibody response 10 to 14 days after the first infection, however, in a small number of people, no antibodies are detected. The question arises about long-term immunity, since there is still insufficient information on the duration of the antibody response. When studying other coronaviruses, it has been shown that the antibody response decreases with time and that infection by homologous coronaviruses can occur 80 days after the first infection, making it a possible the scenario of reinfection, mainly in patients with mild symptoms during the first infection [30,31,32]. It has been shown in a couple of articles that immunoglobulins can decrease to almost half of initial values 36 days after recovery, raising the possibility, in certain cases, that antibodies may not provide prolonged immunity [33,34].

According to PAHO/WHO, in the report issued on October 2020 on interim guidance for the detection of reinfection cases of SARS-CoV-2, they proposed interim criteria and definitions of reinfection cases, based on available information and subject to periodic review. It is concluded that a suspected case of SARS-CoV-2 reinfection is “that symptomatic or asymptomatic person testing positive for SARS-CoV-2, after a period ≥90 days following the first SARS-CoV-2 infection, in whom prolonged excretion of the virus or its RNA and infection by another agent have been ruled out”. It also included the criteria of “a time in which the case was free of symptoms of primary SARS-CoV-2 infection or existence of a time in which the case did not excrete SARS-CoV-2 or viral RNA or existence of negative laboratory testing for SARS-CoV-2 or viral RNA” [35].

In the cases of probable reinfection described in the literature, cases of second infection with practically no symptoms have been found, suggesting a response of the immune system according to the previous encounter; and second cases of greater severity, even fatal, have also been shown, suggesting that the immune system was seriously compromised. Therefore, it has been pointed out that the severity of the reinfection depends on the immune response of each individual, as well as on the dose of virus to which they are exposed, the differences between the SARS-CoV-2 variants and the previous health status [15,36,37].

Cases of probable reinfection have also been reported in healthcare workers, who are highly exposed and in contact with patients with SARS-CoV-2 infection; where age, comorbidities and high exposure are considered to increase their risk of reinfection. However, some reported cases do not meet WHO recommendations for consideration of reinfection. A 24-year-old nurse with no history of pathology, with a 38-day difference between cases and with positive RT-PCR at the onset of symptoms in both cases [31]. Another report showed 2 cases of physicians in patient care units with COVID-19, with a difference of 46 and 24 days between the first and second case, both clinically and by laboratory discharged, and with negative serology for immunoglobulin G (IgG) and immunoglobulin M (IgM) after the first case, which could mean the absence of neutralizing antibodies, which means that the individual did not have the capacity to prevent a subsequent infection by the same agent [15,33]. 

In contrast to our results, it has been shown that SARS-CoV-2 RNA can last in the nasopharynx from 22.7 to 33.5 days in moderate and severe cases respectively, with a maximum of 50 days, which is why, some authors propose the possibility of a reactivation or relapse, since the cases shown in the literature do not provide sufficient evidence to support the theory of a reinfection. According to the authors, the reported cases focus on recurrence of disease observed clinically after initial recovery from COVID-19 infection, others on the timing of test positivity, and in a few other cases a positive test result was obtained following one or two previous negative tests, with patients showing no residual symptoms. Their argument for rejecting the possibility of reinfection is based on the fact that none of the reported cases has a difference of at least 90 days between one episode and another. Some possibilities for this event are considered: first, the presence of a secondary infection in the period where traces of viral RNA can still be found; second, the possibility of an inflammatory rebound due to an inappropriate immune response, which produces recurrence of clinical symptoms; third, prolonged viral persistence, sometimes accompanied by a false negative result in RT-PCR after the first episode; fourth, reactivation of the virus associated with risk factors such as host immunity, virological factors, type and degree of immunosuppression [11,12,13,38,39]. The scope of our work lies in the fact that we show cases that on average had a difference of at least 90 days, or more, between each episode.

As for the reports that support the possibility of reinfection, they consider as a pillar the fact that there are patients with clinical, radiological and laboratory criteria for reinfection, since patients present symptoms, even more severe than the previous case, with positive RT-PCR for SARS-CoV-2 after resolving a first case and with an intermediate negative RT-PCR. Some limitations include the lack of studies regarding the genome of the virus between each case to identify whether the infection was caused by the same virus or a variant of the original one. It is also necessary to study the type of immunological memory developed and its duration. A complete evaluation must be performed, including viral load, a follow-up with detailed clinical evaluation of the patients when a reinfection is suspected [40,41,42,43,44]. 

## 5. Conclusions

The four cases discussed in this article, in health care workers who presented two SARS-CoV-2 infections with a long time free of infection between each episode, confirmed by RT-PCR, provide strong clinical, epidemiological and laboratory evidence to consider the presence of clinical reinfection.

## Figures and Tables

**Table 1:** Sociodemographic, clinical and treatment characteristics of the four cases analyzed in this study.

	Case 1		Case 2		Case 3		Case 4	
Age in years	40		49		53		52	
Gender	Female		Female		Male		Male	
Occupation	Nurse		Nurse		Pharmacy assistant		Internist	
Comorbidities	Yes		No		No			
	First Infection	Second Infection	First Infection	Second Infection	First Infection	Second Infection	First Infection	Second Infection
Onset of symptoms	26 April 2020	07 September 2020	10 May 2020	16 September 2020	22 May 2020	06 September 2020	03 June 2020	24 August 2020
Symptom remission	22 May 2020	10 October 2020	30 May 2020	21 September 2020	20 June 2020	15 October 2020	18 June 2020	17 November 2020
Time between the onset of the first and second infection	134 days		129 days		107 days		82 days	
Time between remission of first infection and onset of second infection	108 days		109 days		78 days		67 days	
Symptoms								
Fever	Yes	Yes			Yes	Yes		
Dry cough	Yes	Yes	Yes	Yes		Yes	Yes	Yes
Headache	Yes	Yes	Yes	Yes				
Rhinorrhea	Yes	Yes				Yes		
General malaise		Yes			Yes	Yes	Yes	Yes
Anosmia	Yes		Yes			Yes		
Chills			Yes			Yes		
Odynophagia				Yes			Yes	Yes
Dyspnea	Minimum efforts	Medium efforts		Medium efforts	Medium efforts			Minimum efforts
Myalgia	Yes	Yes	Yes	Yes				Yes
Arthralgia	Yes	Yes	Yes	Yes				Yes
Decrease in oxygen saturation	Yes	Yes						Yes
Exanthema	Maculopapular on upper and lower limbs, thorax, face and neck		Diffuse fine maculopapular					
Dysgeusia	Yes		Yes			Yes		
Treatment	Paracetamol, nebulizations with budesonide plus ipratropium bromide, salmetol/fluticasone, salbutamol spray, loratadine.	Paracetamol, Salmeterol/ fluticasone, salbutamol spray, montelukast.	Paracetamol y azithromycin.	Budesonide/ formoterol and paracetamol.	Azithromycin, oseltamivir, paracetamol, prednisone, hydroxychloroquinine.	Azithromycin, ivermectin, paracetamol, benzonatate beads, prednisone, indomethacin, beclomethasone spray.	Lopinavir/ritonavir, dexamethasone, azithromycin.	Tocilizumab, linezolid, piperacillin/ tazobactam, enoxaparin, methylprednisolone, dexmedetomidine.
Pneumonia on the second event identified by imaging.		Yes						Yes
Supplemental oxygen requirement	Yes	Yes						Yes
Hospital stay		Yes						Yes
Intensive care unit (ICU) stay								Yes
Assisted mechanical ventilation								Yes (11 days)
Clinical course	Exacerbation/Improvement	Discharged/Pulmonary sequela with postinfectious bullae	Improvement	Improvement	Improvement	Improvement	Improvement	Bilateral interstitial pneumonia/Improvement
